# From single to dual platelet inhibition FIBTEM assay: fibrinogen replacement thresholds for critical bleeding after trauma

**DOI:** 10.1007/s00068-026-03250-0

**Published:** 2026-06-18

**Authors:** Akmez Latona, James Winearls, Alan Ho, Biswadev Mitra

**Affiliations:** 1 Emergency Department, Ipswich Hospital, Ipswich, QLD Australia; 2 LifeFlight Retrieval Medicine, Toowoomba, QLD Australia; 3 School of Public Health and Preventive Medicine, Monash University, Melbourne, VIC Australia; 4 Faculty of Medicine, University of Queensland, Brisbane, QLD Australia; 5 Intensive Care Unit, Gold Coast University Hospital, Gold Coast, QLD Australia; 6 Queensland Cyber Infrastructure Foundation, Brisbane, QLD Australia; 7 Emergency & Trauma Centre, Alfred Health, Melbourne, VIC Australia

**Keywords:** Thrombelastography, Cytochalasin D, Tirofiban, Fibrinogen, Major trauma

## Abstract

**Purpose:**

The FIBTEM assay in Rotational Thromboelastometry (ROTEM^®^) measures fibrin-based clot formation. The reagent transitioned from single to dual-platelet inhibition with addition of tirofiban to cytochalasin D, offering more complete platelet suppression. The aim of this study was to assess the impact of this change on transfusion thresholds in trauma.

**Methods:**

In this retrospective cohort study, we included adult patients with major trauma across Queensland who presented between 2022 and 2025 and had results available for paired ROTEM and conventional coagulation tests on arrival. Patients were sub-grouped into single and dual-platelet inhibition assay groups. Correlations between FIBTEM-A5 and Clauss fibrinogen were analysed, comparing correlation strength and diagnostic accuracy for fibrinogen levels of < 2.0 g/L.

**Results:**

The relationship between FIBTEM-A5 and Clauss fibrinogen was similar using single platelet inhibition assays (R² 0.61;95%CI: 0.51–0.70) and dual-platelet inhibition assays (R² 0.70;95%CI: 0.62–0.77; *p* = 0.94). Predicted FIBTEM-A5 amplitudes at 2 g/L fibrinogen were 7.83 mm (95%CI: 7.42–8.23) and 7.54 mm (95%CI: 7.22–7.87). Diagnostic performance for detecting fibrinogen < 2.0 g/L was comparable between assays, for both FIBTEM-A5 ≤ 10 mm and FIBTEM-A5 ≤ 8 mm (all *p* > 0.05). For the dual-platelet inhibition assay, sensitivity and specificity were 97.8% and 48.1% at ≤ 10 mm, and 92.1% and 74.7% at ≤ 8 mm.

**Conclusion:**

Dual-platelet inhibition did not alter the relationship between FIBTEM-A5 and Clauss fibrinogen or the diagnostic accuracy of FIBTEM thresholds for identifying hypofibrinogenemia. FIBTEM-A5 ≤ 10 mm maintained high sensitivity, while ≤ 8 mm demonstrated greater specificity. Recalibration of established FIBTEM thresholds is not required following transition to dual-platelet inhibition.

**Supplementary Information:**

The online version contains supplementary material available at 10.1007/s00068-026-03250-0.

## Introduction

The initial management of critical bleeding after trauma is commonly guided by viscoelastic haemostatic assays (VHA) [1]. In Queensland, Australia, rotational thromboelastometry (ROTEM^®^; Werfen, Munich, Germany) is the predominant platform, with ROTEM sigma devices used across Queensland Health [2]. VHA-guided resuscitation strategy for shocked trauma patients has been associated with lower transfusion requirements [3]. Current evidence remains heterogenous. Smaller randomised trials have demonstrated a mortality benefit [4–9] whereas, whereas larger trials, such as ITACTIC, did not demonstrate improved survival [10]. It is likely, that the actions from VHA testing need to be optimised, as a test itself does not have capacity to alter outcomes. International guidelines strongly recommend (1 A-B) the use of VHA for coagulation assessment to guide management for critical bleeding after trauma [11, 12].

A key objective of VHA testing is to measure fibrinogen function. During coagulation, fibrinogen is converted into insoluble fibrin by thrombin. The fibrin monomers then polymerize to form strands, which aggregate and branch into a three-dimensional mesh. This mesh acts as a physical scaffold that holds a platelet plug together. Clot formation, structure, and stability are strongly influenced by the conditions present during fibrin generation, including platelet count and function [13]. In ROTEM, the FIBTEM assay isolates this function by inhibiting platelet contribution to clot firmness. A FIBTEM A5 value (amplitude 5 min after clotting time) below 10 mm serves as the fibrinogen replacement threshold during management of critical bleeding after trauma [14].

The FIBTEM assay reagents transitioned from a single to a dual-platelet inhibition formulation in June 2023. The original single assay used Cytochalasin D, which inhibits platelet actin polymerisation but does not fully suppress platelet–fibrin interactions. The dual formulation incorporates Tirofiban, a glycoprotein IIb/IIIa receptor antagonist, providing a more complete inhibition of platelet aggregation and ensuring that FIBTEM A5 reflects fibrin-dependent clot formation alone [15]. The fibrinogen replacement threshold currently applied for trauma haemorrhage within Queensland Health was validated using single-platelet inhibition assay, and the impact of the dual-platelet inhibition formulation on established fibrinogen thresholds was unknown. Therefore, we compared single and dual-platelet inhibition FIBTEM assays in patients with major trauma, to investigate the impact on fibrinogen replacement threshold.

## Methods

### Design

This was a retrospective cohort study using registry and laboratory data from patients who presented to Emergency Departments (EDs) with major trauma across Queensland, Australia, between January 2022 and April 2025.

### Setting and data sources

Queensland has 122 public hospitals with more than 1.8 million Emergency Department (ED) presentations annually, operating across multiple electronic medical record systems. The Queensland Trauma Data Collection (QTDC) serves as the statewide trauma registry, integrating data from all hospitals through structured digital feeds, advanced analytical tools and machine learning algorithms for real-time trauma case detection [16]. Data are sourced from the Emergency Department Information System (EDIS), FirstNet (Cerner), Hospital Based Corporate Information System (HBCIS), Pathology, and Intensive Care Unit systems (Metavision). The registry captures patient demographics, injury characteristics, clinical outcomes, and limited coagulation data (INR). QTDC includes all patients of any age admitted with an Injury Severity Score > 12 (definition of major trauma) or who died following injury, while excluding patients who sustained iatrogenic injuries, isolated neck of femur fractures, injuries secondary to pathological conditions (e.g., pathological fractures due to bony tumours or metastases without a high-energy trauma mechanism), and deaths where injury was not the primary cause.

AUSLAB database is the central laboratory information system for Queensland Health and records conventional coagulation tests (CCT) and VHA results across all participating hospitals. All Queensland Health public hospitals are serviced by Pathology Queensland, a statewide laboratory network. Clauss fibrinogen testing was performed using the von Clauss Measurement System and HemosIL Q.F.A. Thrombin reagent. The same Clauss fibrinogen methodology, reagents, calibration procedures, and quality assurance processes were employed across all Pathology Queensland laboratories.

### Study population

Patients were included if they presented to an emergency department with major trauma and had paired ROTEM and standard coagulation profiles performed on arrival. Pairing was defined as both tests being performed within 30 min of each other, to account for geographical variations in VHA device locations [2]. Only the first ROTEM performed on arrival was included in the analysis. Patients without paired results, or where ROTEM testing was performed beyond the initial ED presentation, were excluded.

Patients were categorised into a single-platelet inhibition assay group (tests before June 2023) and a dual-platelet inhibition assay group (tests after June 2023). The timing of assay transition varied slightly between hospitals as existing stock was used before adopting the dual assay. During this transition period, site-specific laboratory records were reviewed to determine the exact cartridge changeover date for each hospital, and tests were assigned to the corresponding assay group.

### Data variables

Demographic and trauma-related variables were extracted, including age, sex, vital signs on arrival to ED and ISS. ROTEM variables were FIBTEM A5, FIBTEM A10 and EXTEM A10. CCT variables included Clauss fibrinogen (Fib-C), INR and platelet count. While studies have validated the use of A5 over A10 for faster decision-making in trauma resuscitation, we have included FIBTEM A10 in our study, as some hospitals may still be using A10 [17].

### Outcomes

The primary outcome was the association between FIBTEM and laboratory fibrinogen concentrations, examined across the two assay methods to characterise the mechanistic effect of the transition in platelet inhibition. Secondary outcomes included the diagnostic performance of FIBTEM A5 and A10 thresholds for identifying patients with fibrinogen concentrations < 2.0 g/L.

### Statistical analysis

For baseline characteristics, continuous variables were summarised as mean (SD) or median (IQR) and compared using the Welch two-sample t-test. Categorical variables were compared using Pearson’s χ² test. Associations between viscoelastic and laboratory parameters were assessed using linear regression, summarsing the strength of the association using slopes and coefficients of determination (R²). Differences between VHA periods were tested using an interaction between the laboratory parameters and VHA period. Linear regression models were used to estimate predicted FIBTEM A5 and A10 amplitudes at a fibrinogen concentration of 2.0 g/L. We also assessed whether platelet counts modified the relationship between the single- and dual-inhibition assays.

Diagnostic performance for fibrinogen < 2 g/L was evaluated using sensitivity, specificity, predictive values, and the Youden index. These measures were compared between assay groups using the chi-square test. The discriminative ability of VHA methods was also assessed using receiver operating characteristic curves and logistic regression models using an interaction between FIBTEM amplitudes and VHA method.

Statistical significance was defined as two-sided *p* < 0.05. All analyses were performed using R v4.4.1 (Core Team, 2024).

The study was approved by Metro South Human Research Ethics Committee (HREC/2025/QMS/116509). The requirement to seek informed consent from patients was waived. The study was conducted in accordance with the Declaration of Helsinki and the Australian National Statement on Ethical Conduct in Human Research.

## Results

### Study cohort

**Fig. 1:**
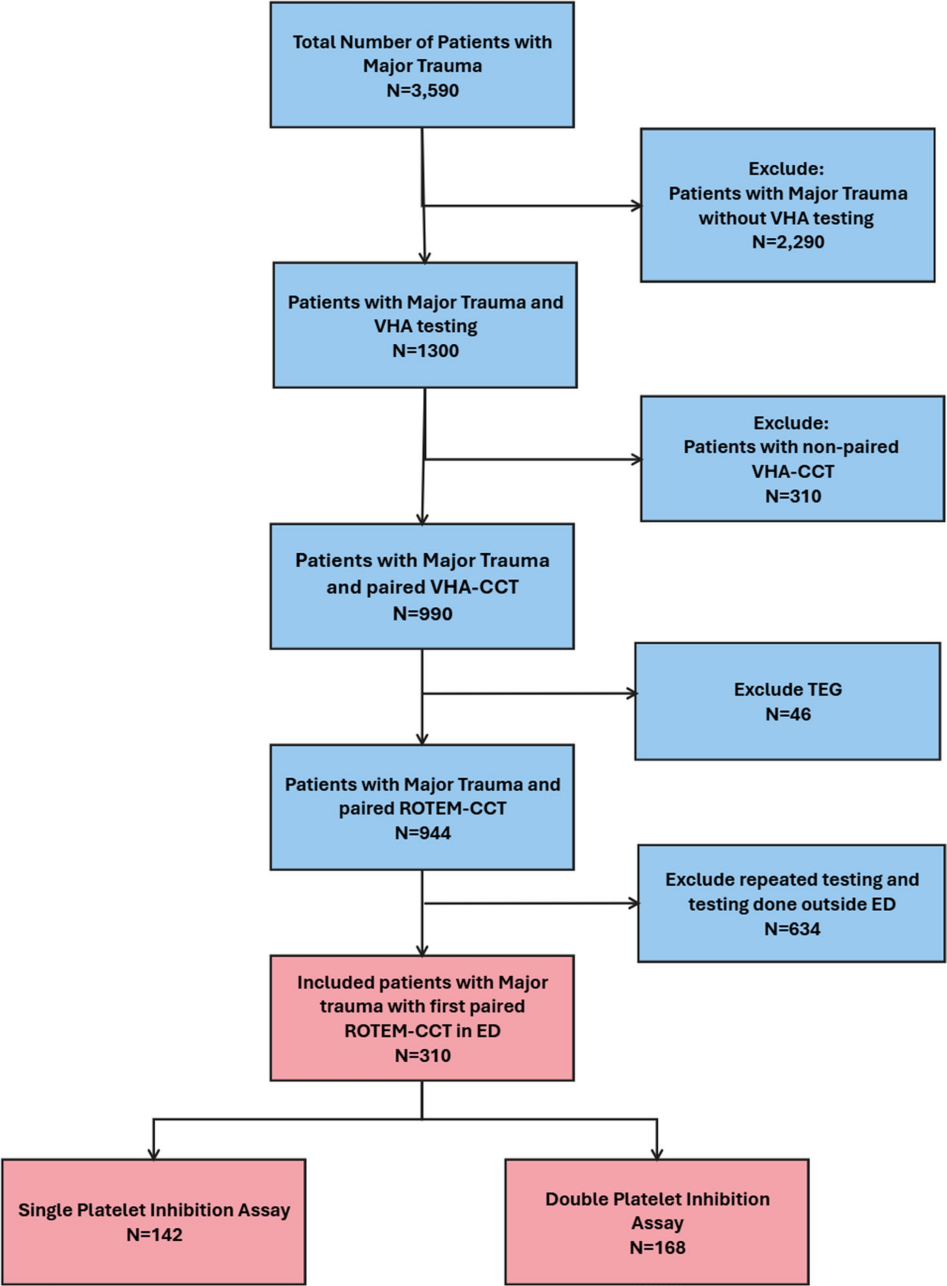
Study flowchart

The inclusion of patients is illustrated in Fig. 1. During the study period, 310 patients met the inclusion criteria and were categorised into single-platelet inhibition assay group (*n* = 142) and dual-platelet inhibition assay group (*n* = 168). Baseline characteristics are presented in Table 1.

**Table 1:** Baseline characteristics of included patients

Characteristic	Overall *N* = 310	Single-platelet inhibition *N* = 142	Dual-platelet inhibition *N* = 168	*p*-value
**Demographics**				
**Age in years** Mean (SD)	41 (20)	40 (22)	42 (20)	0.5^a^
Gender*				> 0.9^c^
Female	61 (20%)	28 (20%)	33 (20%)	
Male	246 (80%)	114 (80%)	132 (80%)	
**Injury Severity Score** Median (IQR)	26 (17–34)	25 (17–33)	26 (19–36)	0.2^b^
**Mechanism of Injury**				0.8c
Blunt	295 (95%)	135 (96%)	160 (95%)	
Penetrating	14 (4.5%)	6 (4.3%)	8 (4.8%)	
**Hospital length of Stay in days** Median (IQR)	11 (3–24)	9 (2–23)	13 (4–25)	0.2^b^
**In-Hospital Death**	18 (5.8%)	11 (7.7%)	7 (4.2%)	0.2c
**Vital Signs**				
**Systolic Blood Pressure (mmHg)** Mean (SD)	111 (29)	108 (26)	114 (30)	0.045^a^
**Heart Rate (bpm)** Mean (SD)	105 (30)	108 (33)	102 (26)	0.080^a^
**Glasgow Coma Scale** Median (IQR)	15.0 (13.0–15.0)	14.0 (12.0–15.0)	15.0 (13.0–15.0)	0.071^b^
**Coagulation and Clot Parameters**				
**INR**	1.20 (SD 0.41)	1.23 (SD 0.52)	1.18 (SD 0.30)	0.3^a^
**Fibrinogen Level** (g/L)	1.96 (SD 0.80)	1.91 (SD 0.77)	2.01 (SD 0.84)	0.3^a^
**FIBTEM A5** (mm)	7.5 (SD 3.8)	7.5 (SD 3.8)	7.6 (SD 3.9)	0.8^a^
**FIBTEM A10** (mm)	8.3 (SD 4.2)	8.3 (SD 4.2)	8.4 (SD 4.3)	0.8^a^

^a^Welch Two Sample t-test

^b^Wilcoxon Rank Sum test

^c^Pearson’s chi-square test; *Missing data for gender, *n* = 3

Fib-C: Clauss Fibrinogen, INR: International Normalised Ratio, FIBTEM: Fibrin-based Thromboelastometric assay, A5: clot amplitude at 5 min, A10: amplitude at 10 min

### Correlation by assay type

For FIBTEM A5 with Fib-C (Fig. 2a), the single-platelet inhibition assay had an R² of 0.61 (95% CI:0.51–0.70) with a slope of 3.88 mm per 1 g/L fibrinogen, while the dual-platelet assay showed an R² of 0.70 (95%:CI 0.62–0.77) with a slope of 3.86 mm per 1 g/L. No significant difference was found between slopes (slope difference = − 0.02; 95% CI − 0.7–0.6; *p* = 0.944).

For FIBTEM A10 with Fib-C (Fig. 2b), R² values were 0.62 (95% CI 0.53–0.72) and 0.71 (95% CI 0.64–0.78) for the single- and dual-platelet assays, respectively, with slopes of 4.29 and 4.30 mm per 1 g/L fibrinogen (slope difference = 0.02; 95% CI − 0.7–0.7; *p* = 0.963).

**Fig. 2a:**
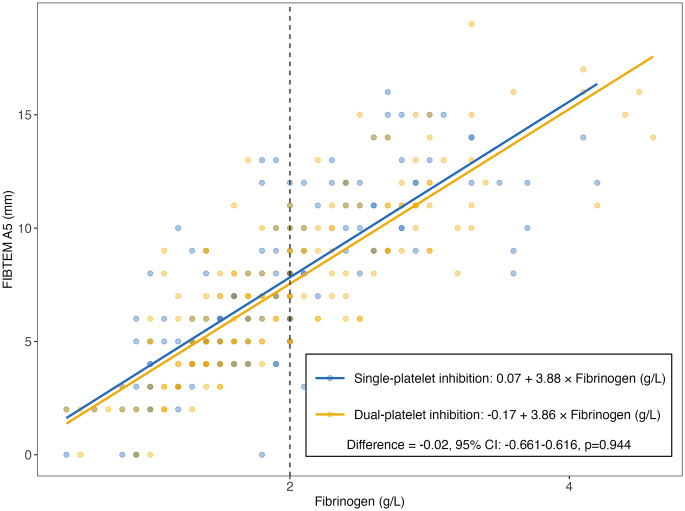
Correlation between FIBTEM A5 vs. Fib-C between Single-platelet vs. Double-platelet inhibition

**Fig. 2b:**
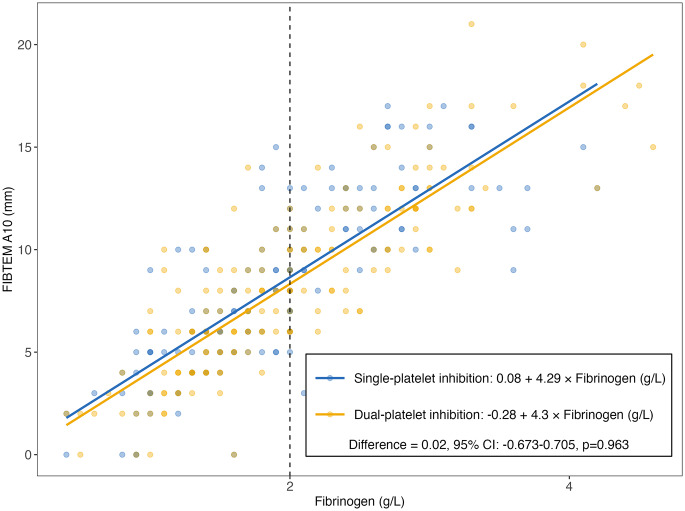
Correlation between FIBTEM A10 vs. Fib-C between Single-platelet vs. Double-platelet inhibition

### Linear regression by assay type

Results of the linear regression between ROTEM and laboratory parameters are presented in Supplementary Material, Table 1. At a fibrinogen concentration of 2 g/L, predicted FIBTEM A5 amplitudes were 7.83 mm (95% CI 7.42–8.23) for the single- and 7.54 mm (95% CI 7.22–7.87) for the dual-platelet assay. Corresponding FIBTEM A10 amplitudes were 8.65 mm (95% CI 8.22–9.08) and 8.33 mm (95% CI 7.97–8.68), respectively. Addition of platelet count as a covariate did not substantially alter the association between Clauss fibrinogen and FIBTEM A5/A10 or materially affect the interaction terms between Clauss fibrinogen and VHA period (Supplementary Table 4).

### Diagnostic accuracy at FIBTEM A5/A10 ≤ 10 mm

For FIBTEM A5 ≤ 10 mm, the dual assay demonstrated similar sensitivity (0.962 vs. 0.978, *p* = 0.55), negative predictive values (NPV) (0.91 vs. 0.95, *p* = 0.51), specificity (0.48 vs. 0.48, *p* = 0.97) and positive predictive value (PPV) (0.68 vs. 0.69, *p* = 0.81) (Supplementary Material, Table 2). Two-by-two contingency tables for FIBTEM A5 thresholds are presented in Table 2.

**Table 2:** Two-by-two contingency tables for FIBTEM A5 and A10 ≤ 10 mm thresholds for identifying fibrinogen concentrations < 2.0 g/L by assay type

Assay Group	Threshold	Fib-C < 2.0 g/L	Fib-C ≥ 2.0 g/L	Total
**FIBTEM A5**				
Single-platelet inhibition	≤ 10 mm	75 (96.2%)	33 (51.6%)	108
	> 10 mm	3 (3.8%)	31 (48.4%)	34
	Total	78 (100%)	64 (100%)	142
Dual-platelet inhibition	≤ 10 mm	87 (97.8%)	41 (51.9%)	128
	> 10 mm	2 (2.2%)	38 (48.1%)	40
	Total	89 (100%)	79 (100%)	168
**FIBTEM A10**				
Single-platelet inhibition	≤ 10 mm	74 (94.9%)	24 (37.5%)	98
	> 10 mm	4 (5.1%)	40 (62.5%)	44
	Total	78 (100%)	64 (100%)	142
Dual-platelet inhibition	≤ 10 mm	85 (96.6%)	35 (44.3%)	120
	> 10 mm	3 (3.4%)	44 (55.7%)	47
	Total	88 (100%)	79 (100%)	167

Fib-C: Clauss Fibrinogen, FIBTEM: Fibrin-based Thromboelastometric assay

For FIBTEM A10 ≤ 10 mm, the dual assay demonstrated similar sensitivity (0.95 vs. 0.97, *p* = 0.58) and NPV (0.91 vs. 0.94, *p* = 0.63), specificity (0.625 vs. 0.557, *p* = 0.411) and PPV (0.76, vs. 0.71, *p* = 0.44) (Supplementary Material, Table 3). Two-by-two contingency tables for FIBTEM A10 thresholds are presented in Table 2.

Receiver operating characteristic analysis demonstrated comparable discrimination for identifying fibrinogen < 2.0 g/L, with AUCs of 0.90 and 0.92 for the single- and dual-platelet inhibition assays, respectively (Supplementary Fig. 1). Logistic regression demonstrated no interaction between assay type and FIBTEM A5 (OR 0.998, 95% CI 0.977–1.020, *p* = 0.862) or A10 (OR 1.000, 95% CI 0.981–1.019, *p* = 0.972), indicating similar prediction of fibrinogen < 2.0 g/L between the single- and dual-platelet inhibition assays (Supplementary Table 5).

### Clinical confidence in FIBTEM A5/A10 thresholds for fibrinogen < 2.0 g/L

Figure 3a illustrates clinical confidence in FIBTEM A5 thresholds for identifying low fibrinogen (< 2.0 g/L). A screening threshold of ≤ 10 mm showed comparable accuracy, with sensitivity 96.2% vs. 97.8% (*p* = 0.55) and specificity 48.4% vs. 48.1% (*p* = 1.00) for the single and dual assays, respectively. A confirmatory threshold of 8 mm maintained high rule-in accuracy, with sensitivity 88.5% vs. 92.1% (*p* = 0.42) and specificity 71.9% vs. 74.7% (*p* = 0.71). No significant differences in rule-in or rule-out performance were observed between assays.

Figure 3b illustrates clinical confidence in FIBTEM A10 thresholds for identifying low fibrinogen (< 2.0 g/L). Figure 3b illustrates FIBTEM A10 thresholds for detecting low fibrinogen (< 2.0 g/L). A screening threshold of ≤ 10 mm demonstrated high rule-out accuracy, with sensitivity 94.9% vs. 96.6% (*p* = 0.58) and specificity 62.5% vs. 55.7% (*p* = 0.41). A confirmatory threshold of 8 mm maintained strong rule-in accuracy, with sensitivity 82.1% vs. 86.4% (*p* = 0.45) and specificity 84.4% vs. 79.7% (*p* = 0.48). No significant differences in rule-in or rule-out accuracy were identified between assays.

**Fig. 3a:**
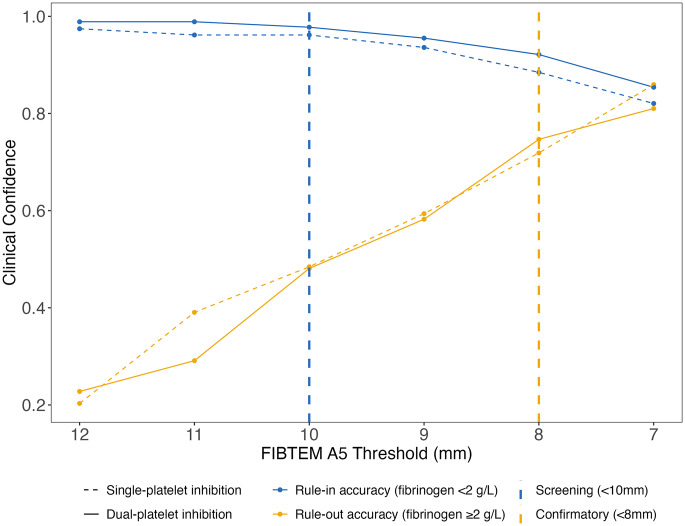
Clinical confidence by FIBTEM A5 (mm) threshold for identifying low fibrinogen (< 2.0 g/L). The blue line represents rule-in accuracy (sensitivity), while the yellow line represents rule-out accuracy (specificity) across different FIBTEM A5 thresholds

**Fig. 3b:**
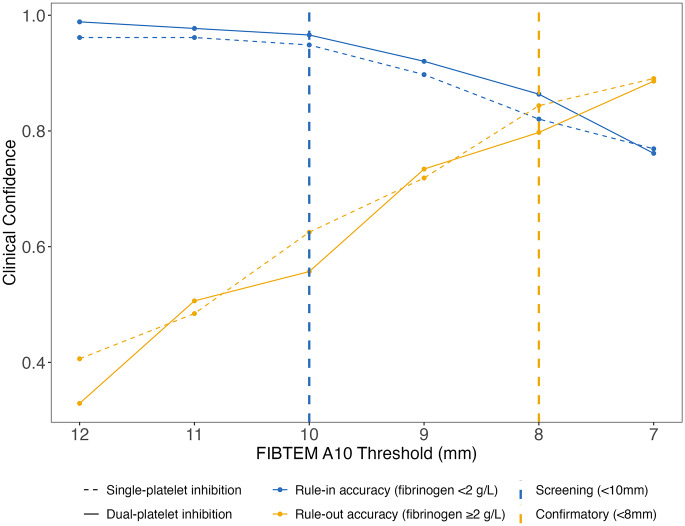
Clinical confidence by FIBTEM A10 (mm) threshold for identifying low fibrinogen (< 2.0 g/L). The blue line represents rule-in accuracy (sensitivity), while the yellow line represents rule-out accuracy (specificity) across different FIBTEM A5 thresholds

## Discussion

This was the first analytical study comparing single vs. dual-platelet inhibition assay against laboratory fibrinogen concentrations in patients with major trauma. There was no difference in the association between FIBTEM-A5 and Fib-C. The slope of the fibrinogen-clot amplitude remained consistent, indicating comparable fibrinogen responsiveness across assay types. Diagnostic performance was equivalent at clinically relevant thresholds, with comparable sensitivity, specificity, and predictive values for identifying low fibrinogen or platelet levels. Although previous studies have demonstrated correlations between Clauss fibrinogen concentrations and FIBTEM amplitudes [18, 19], our study extends this literature by demonstrating that, in a major trauma population, transition from single- to dual-platelet inhibition FIBTEM does not require recalibration of established FIBTEM A5 thresholds for identifying hypofibrinogenaemia.

The introduction of dual-platelet inhibition into the FIBTEM assay aimed to improve analytical isolation of fibrin-based clot formation by minimising residual platelet-mediated clot reinforcement. Incomplete platelet inhibition can result in ongoing platelet-fibrin interactions, thereby increasing clot firmness measurements and potentially overestimating fibrin-dependent clot strength [20]. Cytochalasin D inhibits platelet cytoskeletal contraction, whereas glycoprotein IIb/IIIa antagonists inhibit platelet aggregation through blockade of the GP IIb/IIIa receptor [21]. Combined dual inhibition therefore provides more complete suppression of platelet contribution to clot formation and enhances assay specificity for fibrin polymerisation.

The potential problem of incomplete platelet inhibition in the FIBTEM test had been previously described. Schlimp et al. compared different functional fibrinogen polymerisation assays in 12 healthy volunteers and demonstrated that dual platelet inhibition achieved lower maximum clot firmness MCF than single inhibition (9.5 ± 3.2 mm vs. 11.4 ± 3.1 mm, *p* = 0.02) with whole blood. When tested with platelet-rich and platelet poor-plasma, dual-platelet inhibition produced minimal variation in MCF (1–2%), whereas single inhibition demonstrated higher variability (13–23%) [20]. Similarly, Solomon et al. observed lower MCF values with dual inhibition compared with single inhibition in 30 patients undergoing cardiovascular surgery (19 mm vs. 22, *P* = 0.01) [22]. Biolik et al. confirmed using multiplate impedance aggregometry that combined blockade of the glycoprotein IIb/IIIa receptor and cytochalasin D completely suppressed platelet-mediated clot reinforcement [23].

Previous studies have demonstrated that platelet inhibition can influence fibrin polymerisation clot firmness measurements. Ziegler et al. compared ROTEM Sigma and TEG 6s and reported higher functional fibrinogen values with TEG, likely related to differences in platelet inhibition between assays. TEG functional fibrinogen uses abciximab, whereas FIBTEM uses cytochalasin D, considered a more effective platelet inhibitor. Smaller differences between assays were observed in patients with platelet counts < 150 × 10^9^/L, likely reflecting reduced platelet contribution to clot strength in thrombocytopenic patients [24]. In our study, platelet adjustment did not materially alter the association between Clauss fibrinogen and FIBTEM A5/A10 across the single- and dual-platelet inhibition periods. These findings may suggest limited incremental effect of tirofiban beyond cytochalasin D alone for assessment of fibrin-based clot firmness. Future prospective studies are required to further evaluate the effect of dual platelet inhibition on FIBTEM measurements.

In our study, dual-platelet inhibition was not associated with changes to the slope of the coefficients of determination. These findings suggest that while dual-platelet inhibition may enhance analytical specificity by reducing platelet interference [20, 22, 23], it does not materially alter the clinical relationship between viscoelastic parameters and corresponding laboratory measures of fibrinogen levels or platelet count. Collectively, these findings indicate that improved analytical isolation of fibrin polymerisation does not necessitate recalibration of established transfusion thresholds in trauma patients.

Our study demonstrated comparable performance between the two assays for the current FIBTEM A5/A10 transfusion threshold ≤ 10 mm. For detecting low fibrinogen (< 2.0 g/L), both assays showed high sensitivity at the threshold ≤ 10 mm (96–98%) and maintained similar specificity (48%) and predictive values. Similar trends were observed across different FIBTEM A5/A10 transfusion thresholds (supplementary material). Baksaas-Aasen et al. identified 10 mm as the optimal FIBTEM threshold, yielding the largest AUC for detecting fibrinogen < 2.0 g/L (sensitivity 70%, specificity 76%, NPV 88%, PPV 51%), using ROTEM delta prior to FIBTEM assay change. In contrast, our study demonstrated higher sensitivity but lower specificity at the same threshold, compared with the findings of Baksaas-Aasen et al., likely reflecting differences in study populations and clinical context [10]. Our cohort included patients with major trauma (ISS > 12) across both major trauma centres and regional hospitals with VHA access, represented a broader and more heterogeneous clinical population. Fibrinogen is essential for haemostasis and can reach critically low levels in acute haemorrhage [1]. Prioritising a threshold with high sensitivity is essential to avoid missing patients with hypofibrinogenaemia, even at the expense of reduced specificity. Early identification of hypofibrinogenemia and replacement are paramount to restoring clot integrity. Therefore, maintaining a FIBTEM threshold of ≤ 10 mm remains a pragmatic approach.

We added clinical confidence diagrams to illustrate the trade-off between sensitivity and specificity across different FIBTEM thresholds. A higher, more sensitive threshold (≤ 10 mm) and a lower, more specific threshold (≤ 8 mm) were selected to provide a visual understanding of how diagnostic accuracy changes with clinical decision points. Both assays performed similarly across these thresholds, and the addition of dual-platelet inhibition did not meaningfully alter clinical confidence. A FIBTEM A5 threshold ≤ 8 mm offers high sensitivity with higher specificity, making it a reasonable alternative threshold to adopt. Using FIBTEM A5 ≤ 8 mm as a trigger for fibrinogen replacement implies that approximately one in four patients with Clauss fibrinogen > 2 g/L may still receive fibrinogen based on FIBTEM alone. Some hospitals use FIBTEM A5 ≤ 8 mm to define severe fibrinogen dysfunction and to trigger higher-dose fibrinogen replacement [14]. This definition may warrant revision, as thresholds below 8 mm appear to operate primarily as treatment decision points rather than categorical markers of severe dysfunction.

Our study is subject to the inherent limitations of a retrospective design, including the potential for selection and information bias, possible treatment-before-testing effects, and hospital-level clustering. Although the use of statewide linked datasets enabled comprehensive case capture, only patients with paired ROTEM and Clauss fibrinogen measurements were included, likely representing patients with more severe injury and suspected haemorrhage or coagulopathy, which may limit generalisability to the broader trauma population. ROTEM measurements were limited to those obtained on arrival to the emergency department, and clinical outcomes such as transfusion volume or correction of coagulopathy were not analysed. As the assay groups were separated by calendar time, temporal changes in trauma care, transfusion practice, or ROTEM utilisation may have influenced findings. During the study period, ROTEM Sigma software versions 5.0.0 and 5.3.0 were used across Queensland Health. Although no known calibration or algorithmic updates affecting FIBTEM interpretation were identified, the potential influence of software version changes or unrecognised algorithmic modifications on assay behaviour cannot be completely excluded.

## Conclusion

The transition from the single- to dual-platelet inhibition FIBTEM assay was not associated with a differential relationship between viscoelastic and laboratory measures of fibrinogen. Both assays demonstrated similar diagnostic accuracy for detecting hypofibrinogenaemia at clinically relevant thresholds. FIBTEM-A5 ≤ 10 mm maintained high sensitivity, while ≤ 8 mm demonstrated greater specificity. Recalibration of established FIBTEM thresholds for identifying hypofibrinogenaemia in major trauma patients is not required.

## Supplementary Information

Below is the link to the electronic supplementary material.

Supplementary Material 1

## Data Availability

Data beyond those published are not publicly available due to ethical and governance restrictions, including patient privacy and confidentiality requirements. Access may be considered through Queensland Health, subject to relevant human research ethics and data governance approvals.
