# Supplementary material for: From single to dual platelet inhibition FIBTEM assay: fibrinogen replacement thresholds for critical bleeding after trauma
Source: Eur J Trauma Emerg Surg. 2026 Jun 18;52(1):200. doi: 10.1007/s00068-026-03250-0 (PMC13279660; doi:10.1007/s00068-026-03250-0)

**Supplementary Material**

Supplemental Material content includes additional analyses detailing the association between FIBTEM parameters and laboratory fibrinogen concentrations, including linear regression results and diagnostic performance of FIBTEM A5 and A10 thresholds for hypofibrinogenaemia.

Supplementary Material, Table 1. Linear regression analysis by assay type for FIBTEM A5 and FIBTEM A10

| ASSAY | CARTRIDGE | R^2^ (95% CI) | EQUATION | DIFFERENCE IN SLOPES (95% CI) | LEVELS | PREDICTED (95% CI) |
| --- | --- | --- | --- | --- | --- | --- |
| FIBRINOGEN g/L | | | | | | |
| FIBTEM A5 | Single-Platelet Inhibition | 0.61  (0.51 - 0.7) | 0.07 + 3.88X | -0.02 (-0.661- 0.616), p=0.944 | 1 | 3.95  (3.33-4.57) |
|  |  |  |  |  | 1.5 | 5.89  (5.43-6.34) |
|  |  |  |  |  | 2 | 7.83  (7.42-8.23) |
|  | Dual-Platelet Inhibition | 0.7  (0.62 - 0.77) | -0.17 + 3.86X |  | 1 | 3.69  (3.18-4.19) |
|  |  |  |  |  | 1.5 | 5.61  (5.24-5.99) |
|  |  |  |  |  | 2 | 7.54  (7.22-7.87) |
| FIBTEM A10 | Single-Platelet Inhibition | 0.62  (0.53 - 0.72) | 0.08 + 4.29X | 0.02  (-0.673- 0.705), p=0.963 | 1 | 4.37  (3.7-5.03) |
|  |  |  |  |  | 1.5 | 6.51  (6.03-6.99) |
|  |  |  |  |  | 2 | 8.65  (8.22-9.08) |
|  | Dual-Platelet Inhibition | 0.71  (0.64 - 0.78) | -0.28 + 4.3X |  | 1 | 4.02  (3.47-4.58) |
|  |  |  |  |  | 1.5 | 6.17  (5.76-6.59) |
|  |  |  |  |  | 2 | 8.33  (7.97-8.68) |

Supplementary Material, Table 2. FIBTEM A5 thresholds for Fib-C <2.0 g/L

| CARTRIDGE | THRESHOLD | TP / FP / FN / TN | SENSITIVITY  (95% CI) | SPECIFICITY  (95% CI) | POSITIVE PREDICTIVE  VALUE  (95% CI) | NEGATIVE PREDICTIVE  VALUE  (95% CI) | YOUDEN INDEX  (95% CI) |
| --- | --- | --- | --- | --- | --- | --- | --- |
| SINGLE-PLATELET INHIBITION | 12 | 76 / 51 / 2 / 13 | 0.97  (0.94 - 1) | 0.2  (0.11 - 0.3) | 0.6  (0.57 - 0.63) | 0.88  (0.68 - 1) | 0.17  (0.05 to 0.3) |
|  | 11 | 75 / 39 / 3 / 25 | 0.96  (0.91 - 1) | 0.39  (0.27 - 0.52) | 0.66  (0.61 - 0.71) | 0.9  (0.78 - 1) | 0.35  (0.18 to 0.52) |
|  | 10 | 75 / 33 / 3 / 31 | 0.96  (0.91 - 1) | 0.48  (0.36 - 0.61) | 0.69  (0.64 - 0.75) | 0.92  (0.81 - 1) | 0.44  (0.27 to 0.61) |
|  | 9 | 73 / 26 / 5 / 38 | 0.94  (0.87 - 0.99) | 0.59  (0.47 - 0.72) | 0.74  (0.68 - 0.8) | 0.89  (0.79 - 0.97) | 0.53  (0.34 to 0.71) |
|  | 8 | 69 / 18 / 9 / 46 | 0.88  (0.81 - 0.95) | 0.72  (0.61 - 0.83) | 0.79  (0.73 - 0.86) | 0.84  (0.75 - 0.92) | 0.6  (0.42 to 0.78) |
|  | 7 | 64 / 9 / 14 / 55 | 0.82  (0.73 - 0.9) | 0.86  (0.77 - 0.94) | 0.88  (0.81 - 0.94) | 0.8  (0.72 - 0.88) | 0.68  (0.5 to 0.84) |
| DUAL-PLATELET INHIBITION | 11 | 88 / 56 / 1 / 23 | 0.99  (0.97 - 1) | 0.29  (0.2 - 0.39) | 0.61  (0.58 - 0.65) | 0.96  (0.87 - 1) | 0.28  (0.17 to 0.39) |
|  | 10 | 87 / 41 / 2 / 38 | 0.98  (0.94 - 1) | 0.48  (0.38 - 0.59) | 0.68  (0.64 - 0.73) | 0.95  (0.88 - 1) | 0.46  (0.32 to 0.59) |
|  | 9 | 85 / 33 / 4 / 46 | 0.96  (0.91 - 0.99) | 0.58  (0.47 - 0.7) | 0.72  (0.67 - 0.78) | 0.92  (0.84 - 0.98) | 0.54  (0.38 to 0.69) |
|  | 8 | 82 / 20 / 7 / 59 | 0.92  (0.87 - 0.97) | 0.75  (0.65 - 0.84) | 0.8  (0.74 - 0.87) | 0.9  (0.82 - 0.95) | 0.67  (0.52 to 0.81) |
|  | 7 | 76 / 15 / 13 / 64 | 0.85  (0.78 - 0.92) | 0.81  (0.72 - 0.9) | 0.84  (0.77 - 0.91) | 0.83  (0.76 - 0.9) | 0.66  (0.5 to 0.82) |

Supplementary Material, Table 3. FIBTEM A10 thresholds for Fib-C <2.0 g/L

| CARTRIDGE | THRESHOLD | TP / FP / FN / TN | SENSITIVITY | SPECIFICITY | POSITIVE PREDICTIVE VALUE | NEGATIVE PREDICTIVE VALUE | YOUDEN INDEX |
| --- | --- | --- | --- | --- | --- | --- | --- |
| SINGLE-PLATELET INHIBITION | 12 | 75 / 38 / 3 / 26 | 0.96  (0.91 - 1) | 0.41  (0.3 - 0.53) | 0.66  (0.62 - 0.71) | 0.9  (0.78 - 1) | 0.37  (0.21 to 0.53) |
|  | 11 | 75 / 33 / 3 / 31 | 0.96  (0.91 - 1) | 0.48  (0.38 - 0.61) | 0.69  (0.65 - 0.75) | 0.92  (0.82 - 1) | 0.44  (0.29 to 0.61) |
|  | 10 | 74 / 24 / 4 / 40 | 0.95  (0.9 - 0.99) | 0.62  (0.52 - 0.75) | 0.75  (0.7 - 0.82) | 0.91  (0.82 - 0.98) | 0.57  (0.42 to 0.74) |
|  | 9 | 70 / 18 / 8 / 46 | 0.9  (0.82 - 0.96) | 0.72  (0.61 - 0.83) | 0.8  (0.73 - 0.86) | 0.85  (0.77 - 0.94) | 0.62  (0.43 to 0.79) |
|  | 8 | 64 / 10 / 14 / 54 | 0.82  (0.73 - 0.9) | 0.84  (0.75 - 0.92) | 0.87  (0.8 - 0.93) | 0.79  (0.72 - 0.87) | 0.66  (0.48 to 0.82) |
|  | 7 | 60 / 7 / 18 / 57 | 0.77  (0.68 - 0.86) | 0.89  (0.81 - 0.97) | 0.9  (0.83 - 0.96) | 0.76  (0.69 - 0.84) | 0.66  (0.49 to 0.83) |
| DUAL-PLATELET INHIBITION | 11 | 86 / 39 / 2 / 40 | 0.98  (0.94 - 1) | 0.51  (0.41 - 0.61) | 0.69  (0.65 - 0.74) | 0.95  (0.88 - 1) | 0.49  (0.35 to 0.61) |
|  | 10 | 85 / 35 / 3 / 44 | 0.97  (0.92 - 1) | 0.56  (0.46 - 0.67) | 0.71  (0.66 - 0.76) | 0.94  (0.87 - 1) | 0.53  (0.38 to 0.67) |
|  | 9 | 81 / 21 / 7 / 58 | 0.92  (0.86 - 0.98) | 0.73  (0.63 - 0.82) | 0.8  (0.74 - 0.86) | 0.9  (0.82 - 0.96) | 0.65  (0.49 to 0.8) |
|  | 8 | 76 / 16 / 12 / 63 | 0.86  (0.78 - 0.93) | 0.8  (0.71 - 0.89) | 0.83  (0.76 - 0.89) | 0.84  (0.77 - 0.91) | 0.66  (0.49 to 0.82) |
|  | 7 | 67 / 9 / 21 / 70 | 0.76  (0.67 - 0.85) | 0.89  (0.81 - 0.95) | 0.88  (0.81 - 0.95) | 0.77  (0.7 - 0.84) | 0.65  (0.48 to 0.8) |

Supplementary Table 4. Linear regression analysis evaluating the association between Clauss fibrinogen and FIBTEM A5/A10 before and after adjustment for platelet count, comparing the single- and dual-platelet inhibition periods.

|  | **FIBTEM A5** | | **FIBTEM A10** | |
| --- | --- | --- | --- | --- |
| **CHARACTERISTIC** | **BETA**  **(95% CI)** | **P** | **BETA**  **(95% CI)** | **P** |
| Unadjusted for Platelet | | | | |
| Fib C | 3.9  (3.4, 4.4) | <0.001 | 4.3  (3.8, 4.8) | <0.001 |
| VHA Period |  |  |  |  |
| Single-platelet inhibition | — |  | — |  |
| Dual-platelet inhibition | -0.24  (-1.6, 1.1) | 0.7 | -0.36  (-1.8, 1.1) | 0.6 |
| Fib C * VHA Period |  |  |  |  |
| Fib C * Dual-platelet inhibition | -0.02  (-0.66, 0.62) | >0.9 | 0.02  (-0.67, 0.70) | >0.9 |
| Adjusted for Platelet | | | | |
| Fib C | 3.9  (3.4, 4.4) | <0.001 | 4.4  (3.8, 4.9) | <0.001 |
| VHA Period |  |  |  |  |
| Single-platelet inhibition | — |  | — |  |
| Dual-platelet inhibition | -0.19  (-1.5, 1.2) | 0.8 | -0.12  (-1.6, 1.3) | 0.9 |
| Platelet | 0.00  (-0.01, 0.00) | 0.068 | 0.00  (-0.01, 0.00) | 0.024 |
| Fib C * VHA Period |  |  |  |  |
| Fib C * Dual-platelet inhibition | 0.09  (-0.56, 0.74) | 0.8 | 0.06  (-0.63, 0.75) | 0.9 |

Supplementary Table 5. Logistic regression analysis evaluating difference in the relationship between FIBTEM A5 and FIBTEM A10 and VHA Method on predicting Fib C < 2.0.

|  | FIBTEM A5 | | FIBTEM A10 | |
| --- | --- | --- | --- | --- |
| *CHARACTERISTIC* | **OR (95% CI)** | **P** | **OR (95% CI)** | **P** |
| FIBTEM | 0.915  (0.901 to 0.930) | **<0.001** | 0.922  (0.908 to 0.935) | **<0.001** |
| VHA |  |  |  |  |
| Single-platelet inhibition | - |  | - |  |
| Dual-platelet inhibition | 1.003  (0.838 to 1.200) | 0.974 | 0.989  (0.826 to 1.183) | 0.901 |
| FIBTEM x VHA | 0.998  (0.977 to 1.020) | 0.862 | 1.000  (0.981 to 1.019) | 0.972 |

Supplementary Figure 1. Receiver operating characteristic curves for FIBTEM A5 and A10 predicting fibrinogen <2.0 g/L using single-platelet inhibition and dual-platelet inhibition assays.


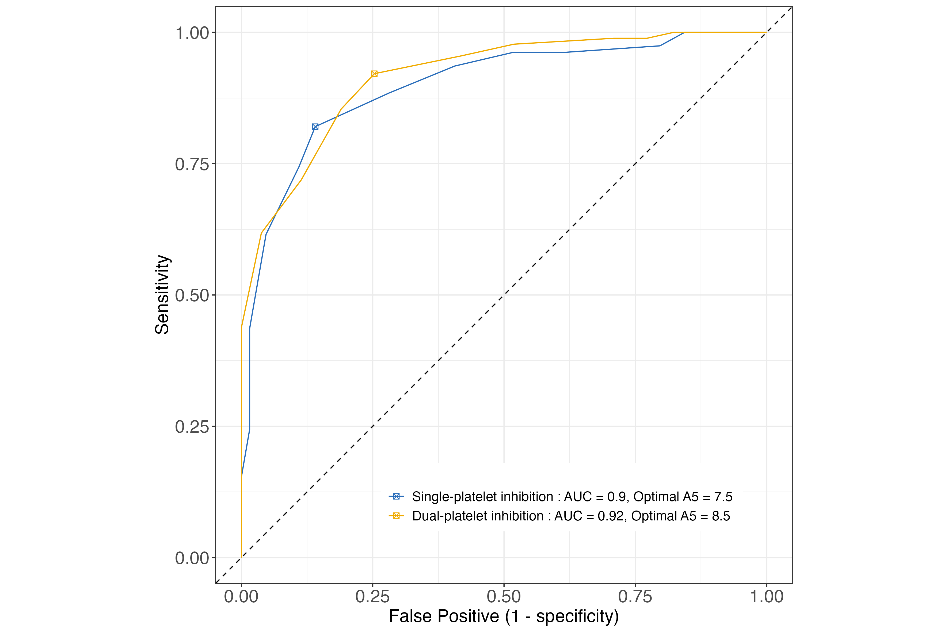

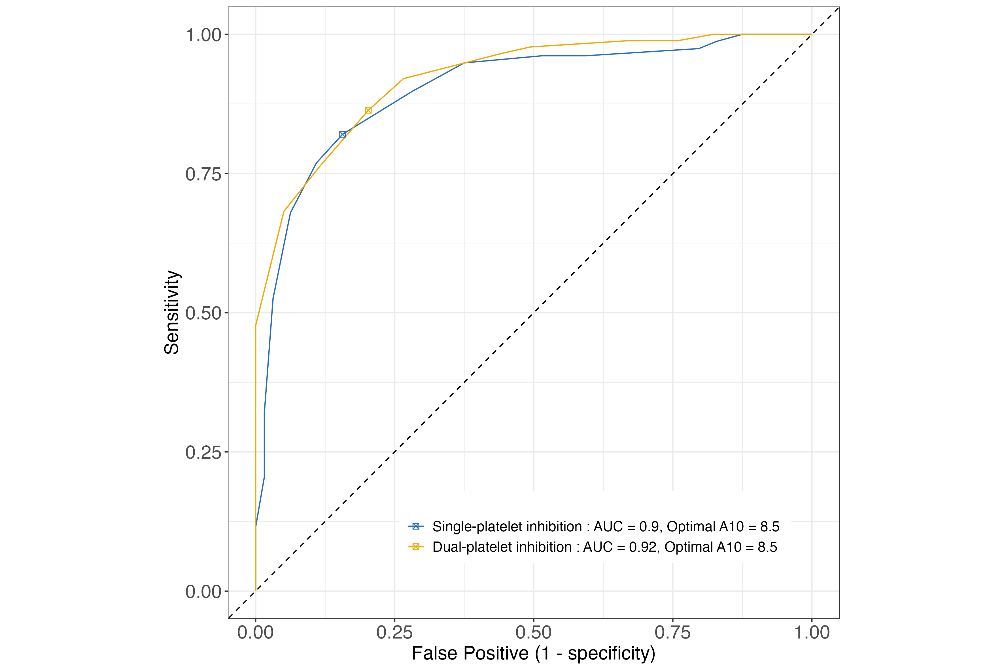

Supplement: Supplementary file 1 — Supplementary Material 1 [file 68_2026_3250_MOESM1_ESM.docx]
